# STAG2 promotes naive-primed transition *via* activating Lin28a transcription in mouse embryonic stem cells

**DOI:** 10.1016/j.jbc.2024.107958

**Published:** 2024-11-05

**Authors:** Bo Chen, Mingkang Jia, Gan Zhao, Yumin Liu, Yihong Song, Mengjie Sun, Wangfei Chi, Xiangyang Wang, Qing Jiang, Guangwei Xin, Chuanmao Zhang

**Affiliations:** 1 The Key Laboratory of Cell Proliferation and Differentiation of the Ministry of Education, College of Life Sciences, Peking University, Beijing, China; 2 The Academy for Cell and Life Health, Faculty of Life Science and Technology, Kunming University of Science and Technology, Kunming, China

**Keywords:** cell cycle, cell differentiation, STAG2, naive-primed transition, Lin28a, mouse embryonic stem cells

## Abstract

Mouse embryonic stem cells (mESCs) exist in two distinct pluripotent states: the naive and the primed. Mainly by inducing differentiation of mESCs *in vitro*, conducting RNA sequencing analyses, and specifying expression of the regulatory genes, we explored the regulatory mechanisms underlying the transition between the naive and primed states. We found that, under the defined differentiation-inducing conditions, the naive state of mESCs shifted to the primed state within 2 days of differentiation induction, during which the cell cycle- and differentiation-related proteins changes significantly. Specifically, we uncovered that the expression of STAG2, a subunit of the Cohesin complex, was upregulated. We further revealed that knockout of STAG2 resulted in upregulation of the naive gene sets and downregulation of the primed gene sets, indicating importance of STAG2 in regulating the naive-primed transition. More importantly, STAG2 knockout led to a reduction in number of the bivalent genes, a decrease in Lin28a transcription, and a reduced cytoplasmic localization of Lin28a. Overexpressing Lin28a or a Lin28a variant lacking the nucleolar localization signal (Lin28aΔNoLS) in STAG2 knockout cells rescued the downregulation of the primed marker genes *Dnmt3a/3b*. Collectively, we conclude that STAG2 facilitates the naive-primed transition of mESCs by activating Lin28a transcription and that this work may offer a new insight into the regulation of pluripotency in mESCs.

Mouse embryonic stem cells (mESCs) are pluripotent stem cells derived from the inner cell mass of blastocysts. These cells can be cultured *in vitro* in defined conditions including with addition of the leukemia inhibitory factor (LIF) (1). During the culture *in vitro*, mESCs exist in two distinct pluripotent states: the naive and the primed, which are respectively corresponding to the pluripotency of the pre-implantation and the post-implantation epiblast cells during embryonic development *in vivo* (2). Naive mESCs require the addition of LIF and two inhibitors (2i), CHIR99021 (an inhibitor of GSK3) and PD032590 (an inhibitor of MEK), to maintain their self-renewal when cultured *in vitro* (3). LIF and 2i activate the JAK/STAT3 signaling pathway (4, 5, 6), inhibit GSK3 activity in the WNT pathway (7, 8, 9), and suppress the FGF4/ERK signaling pathway, thereby promoting self-renewal and preventing differentiation (10, 11, 12). Cells in the primed pluripotent state, known as Epiblast Stem Cells (EpiSCs), generally require Fibroblast Growth Factor 2 (FGF2) and Activin A to maintain their pluripotent state during *in vitro* culture (13, 14).

During the transition from naive to primed pluripotency, mESCs undergo significant epigenetic changes, including DNA demethylation and X chromosome inactivation. Naive pluripotent mESCs possess a hypomethylated genome, whereas primed pluripotent mESCs exhibit a more methylated genome (15, 16, 17). The regulation of DNA methylation is mediated by the action of DNA methyltransferases (Dnmt). Inhibition of MEK and GSK3 results in decreased levels of Dnmt3a/3b, thereby sustaining the low methylation state characteristic of naive pluripotency (16, 18). The RNA-binding protein Lin28a has also been shown to regulate Dnmt3a/3b levels and facilitate the transition from naive to primed pluripotency (19). Moreover, naive and primed pluripotent mESCs express distinct marker genes. Naive mESCs typically express genes such as *Klf4* and *Nanog*, while primed mESCs express genes such as *Fgf5* and *Otx2* (4).

The cell cycle of naive mESCs is approximately 12 h, featuring a shorter G1 phase compared to somatic cells (20). The interplay between cell cycle dynamics and pluripotency factors in mESCs is crucial for determining their self-renewal and differentiation processes (21, 22). The Cohesin complex, a vital regulator of the cell cycle, plays multiple roles including preserving genome stability, facilitating DNA damage repair, and regulating gene expression (23, 24). The Cohesin complex comprises four subunits: SMC1, SMC3, RAD21, and STAG. Among these, the core subunits STAG1 and STAG2, which are mutually exclusive homologues, have been extensively studied for their functional redundancy and distinct roles. The absence of either subunit is lethal at different stages of embryonic development (25, 26, 27, 28). Additionally, *STAG2* is considered an important tumor suppressor gene, with its deletion being associated with various cancers such as glioblastoma multiforme (GBM), Ewing's sarcoma, and melanoma (29). The formation of topologically associating domains (TADs) and the regulation of gene transcription in mESCs are heavily reliant on the essential involvement of STAG1-Cohesin and STAG2-Cohesin, which mediate chromatin loops (28). However, there is a paucity of studies focusing on their roles in embryonic stem cells (ESCs), particularly in relation to the regulation of pluripotency.

Research on the regulation of pluripotency in mESCs has been a prominent and intensely studied field, particularly concerning the transition between naive and primed pluripotency states. The intricate interplay between cell cycle regulators and pluripotency factors in mESCs significantly influences their fate determination during proliferation and differentiation. Consequently, the collaborative roles of the Cohesin complex and pluripotency factors in regulating the conversion between pluripotency states, as well as the underlying molecular mechanisms, remain to be fully elucidated.

## Results

### mESCs undergo a transition from a naive state to a primed state within 2 days under differentiation conditions

In this study, we employed Serum/LIF medium supplemented with 2i as the basal medium for culturing mESCs (Fig. S1, *A–G*) and subsequently transferred them to a medium devoid of LIF and 2i to induce differentiation. Cell samples were collected at 0-, 1-, 2-, 3-, 4- and 5-days post-transfer and designated as S0d, S1d, S2d, S3d, S4d, and S5d, respectively. Each time point included three replicate samples, which were subjected to RNA-seq analysis. Principal component analysis of the sequencing results revealed that the three replicate samples under each treatment condition were highly correlated (data not shown), indicating high reproducibility and reliable data.

**Figure 1:**
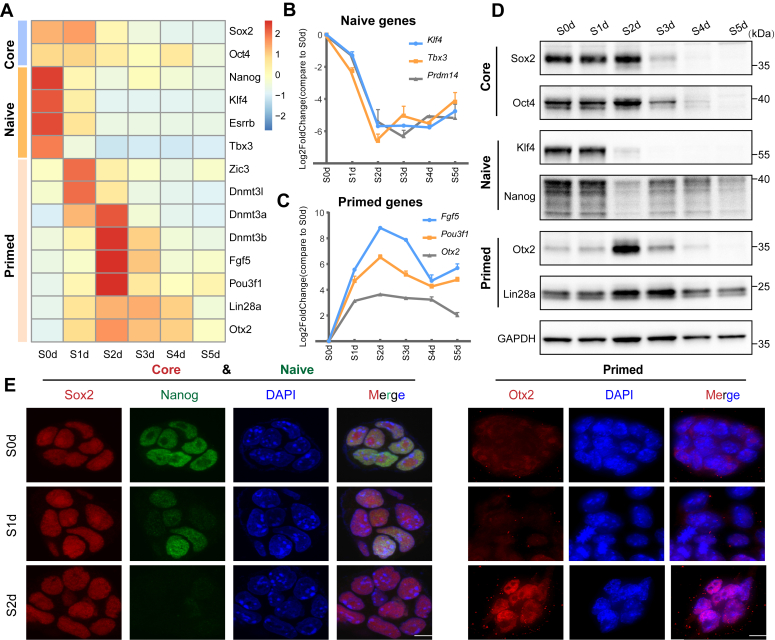
**Naive-primed transition initiation after 2 days of differentiation in mESCs.***A*, heatmap of differentially expressed pluripotency marker genes during differentiation, with *Sox2* and *Oct4* highly expressed in both naive and primed states. Naive marker genes peak at S0d, while primed marker genes peak from S2d onwards. *B* and *C*, expression trends of naive and primed marker genes, respectively, averaged from three replicates with error bars indicating standard deviation. *D*. Western blot analysis of pluripotency marker proteins during differentiation, with GAPDH as the loading control. *E*, immunofluorescence assay detecting the localization and levels of proteins corresponding to pluripotency marker genes in S0d, S1d, and S2d cells, with DAPI staining the nucleus (scale bar: 10 μm).

Through the analysis of RNA-seq data, we identified differentially expressed pluripotency marker genes and performed hierarchical clustering. Our results showed that naive marker genes were highly expressed at S0d and gradually decreased during differentiation, while primed marker genes were highly expressed at S2d and exhibited an increasing-decreasing pattern during differentiation (Fig. 1*A*). To further elucidate the changes in naive and primed marker genes during differentiation, we selected three representative genes and plotted their expression levels at different time points relative to the control group. The results demonstrated that naive marker genes decreased significantly at S2d and exhibited a pattern of rapid decline followed by slow increase (Fig. 1*B*), while primed marker genes showed the opposite pattern, increasing in expression during differentiation and peaking at S2d (Fig. 1*C*).

To validate these findings, we performed Western blotting to detect the protein levels of several genes and found that at S2d, the naive pluripotency factors Nanog and Klf4 were significantly downregulated, while the primed pluripotency factor Otx2 and Lin28a were upregulated and reached their maximum expression, and the core pluripotency factors Sox2 and Oct4 remained highly expressed (Fig. 1*D*). Immunofluorescence staining of mESCs at S0d, S1d, and S2d also showed similar results, with core pluripotency factors Sox2 maintaining high expression, naive pluripotency factors Nanog decreasing in expression, and primed pluripotency factors Otx2 increasing in expression (Fig. 1*E*).

Collectively, our results demonstrate that mESCs exit the naive pluripotency state and undergo a transition from naive to primed pluripotency during the differentiation process from S0d to S2d.

### Differential regulation of STAG2 and STAG1 expression in the naive to primed pluripotency transition

**Figure 2:**
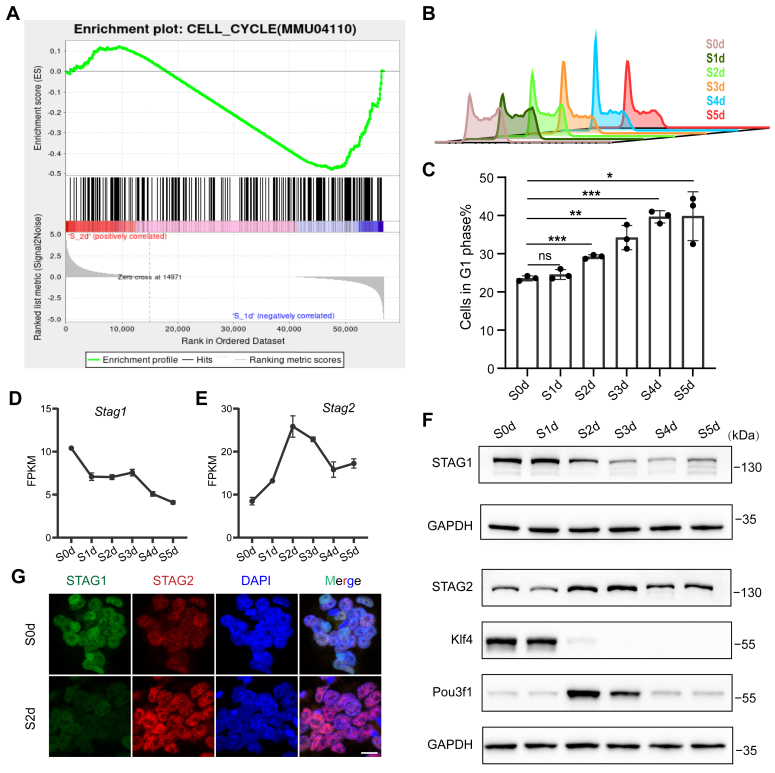
**Downregulation of STAG1 and upregulation of STAG2 during the naive-primed transition in mESCs.***A*, gene Set Enrichment Analysis (GSEA) comparing changes in the cell cycle pathway (KEGG ID mmu04110) between S1d and S2d. *B* and *C*, flow cytometry analysis of the cell cycle in mESCs at different time points of differentiation. Experiments were repeated in triplicate. Results revealed a significant increase in the G1 phase cell population beginning on day 2 (S2d) and persisting through the subsequent 3 days of differentiation. Statistical significance was determined using a two-tailed *t* test (∗∗∗*p* < 0.001). *D* and *E*, changes in the expression levels of Cohesin complex subunits STAG1 and STAG2 genes during differentiation. The Y-axis represents FPKM (Fragments per kilobase of exon model per million mapped fragments). Data are averaged from three replicates, with error bars indicating standard deviation. Error bars are not shown if too small to exceed the size of the node symbol. *F*, Western blot analysis of STAG1 and STAG2 protein levels during differentiation, with GAPDH as the loading control. G. immunofluorescence assay detecting the nuclear localization of STAG1 and STAG2 proteins in S0d and S2d cells, with DAPI staining the nucleus (scale bar: 10 μm).

As previously stated, our results revealed that mESCs exited the naive pluripotency state and underwent a transition to primed pluripotency at S2d of differentiation. Employing gene set enrichment analysis, we discerned differentially expressed gene sets pertinent to biological processes or pathways throughout differentiation. The outcomes demonstrated a noteworthy alteration in the cell cycle pathway at S2d, characterized by the repression of cell cycle genes in contrast to S1d (Fig. 2*A*). Subsequently, employing flow cytometry analysis, we observed a significant increase in the proportion of G1 phase cells from S2d onwards, indicative of the transition from naive to primed pluripotency (Fig. 2, *B* and *C*). This observation underscores the potential involvement of cell cycle regulators in this process.

Analyzing the RNA-seq data, we delineated changes in the transcriptional levels of cell cycle-related genes during the naive-primed transition. Notably, STAG1 gene expression exhibited a gradual decrease, while STAG2 gene expression displayed an increasing-decreasing pattern, peaking at S2d (Fig. 2, *D* and *E*). To corroborate these findings, we examined the protein levels of STAG1 and STAG2 during the transition and observed a significant decrease in STAG1 protein levels at S2d, akin to the pattern observed for the naive pluripotency factor Klf4, while STAG2 protein levels increased significantly at S2d, akin to the primed pluripotency factor Pou3f1 (Fig. 2*F*). Immunofluorescence experiments also confirmed that compared to the naive state(S0d), the expression level of STAG1 decreased on the second day of differentiation (S2d), whereas STAG2 exhibited an opposite expression pattern at this stage (Fig. 2*G*).

Collectively, our investigation highlights the opposing expression patterns of STAG1 and STAG2, core subunits of the Cohesin complex, during the transition from naive to primed pluripotency in mESCs. Specifically, STAG1 expression paralleled that of naive pluripotency factors, whereas STAG2 expression mirrored that of primed pluripotency factors, suggesting their pivotal roles in this pluripotency state transition.

### STAG2 deficiency facilitates the maintenance of naive pluripotency in mESCs

To further elucidate the roles of STAG1 and STAG2 in maintaining pluripotency and orchestrating transitions in mESCs, we employed CRISPR-Cas9 technology to generate STAG1 or STAG2 knockout (KO) mESCs lines (Figs. S2 and S3). Given the pivotal involvement of STAG1 and STAG2 in cell cycle dynamics, we initially assessed the impact of their deletion on cell cycle progression. Our findings indicate that the deletion of either STAG1 or STAG2 minimally affects cell cycle progression during the M phase, with relatively modest effects observed during the S phase (Fig. S4, *A–F*). Notably, while the deletion of STAG1 leads to a significant increase in the proportion of G1 phase cells, STAG2 deletion does not exhibit a substantial alteration in G1 phase cells, resembling characteristics reminiscent of the primed pluripotent state (Fig. S4, *G* and *H*).

To delve deeper into the regulatory roles of STAG1 and STAG2 in pluripotency, we conducted RNA-seq analysis to unveil changes in gene expression profiles in these KO cells. Our results unveil that the deletion of STAG1 results in differential expression of 1278 genes, whereas STAG2 deletion impacts the expression of 629 genes (Fig. S5, *A* and *B*). Functional enrichment analysis underscores the significant impact of STAG1 deletion on the TGF-β signaling pathway and ribosomal RNA (rRNA) processes, aligning with previous findings (30). Conversely, the analysis of STAG2 KO cells reveals enrichment of terms associated with stem cell differentiation, implicating STAG2 in the regulation of pluripotency and differentiation pathways (Fig. S5, *C* and *D*). In exploring the influence of STAG1 and STAG2 on mESCs differentiation, induction of differentiation in STAG1 or STAG2 knockout cell lines revealed distinctive patterns. Notably, the loss of STAG1 predominantly downregulates endoderm marker genes (Fig. S6), while STAG2 deficiency results in varying degrees of downregulation across marker genes for all three germ layers (Fig. S7), suggesting a pivotal role for STAG2 in regulating differentiation across germ layers. Consequently, our subsequent analyses predominantly focus on elucidating the function of STAG2 in mESCs pluripotency regulation.

**Figure 3:**
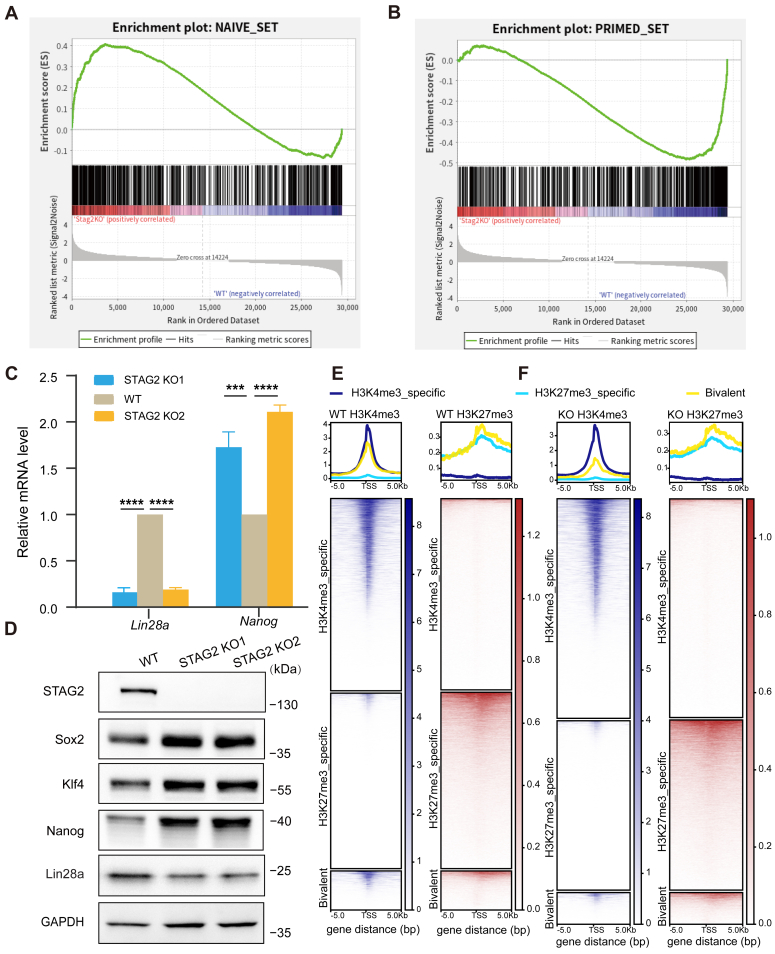
**Loss of STAG2 leads to increased expression of naive marker genes and decreased expression of primed marker genes.***A* and *B*, naive and Primed Gene Set Enrichment Analysis. The naive gene set is relatively highly expressed, while the primed gene set is relatively lowly expressed in STAG2 KO cells compared to WT. Enrichment profile indicates activation or repression of gene sets in STAG2 KO cells. *C*, qPCR analysis of transcriptional changes in *Sox2*, *Nanog*, and *Lin28a* in two STAG2 KO cell lines. Averaging data from three replicates with standard deviation error bars. Statistical significance was determined using two-tailed t-tests: ∗∗∗∗*p* < 0.0001, ∗∗∗*p* < 0.001, ∗∗*p* < 0.01. *D*, Western blot analysis of pluripotency factors in two STAG2 KO cell lines, with GAPDH as the loading control. *E* and *F*, heatmaps of H3K4me3 or H3K27me3 peak distributions in WT and STAG2 KO cells, with gene representation and normalized Reads density indicating protein enrichment across peak distribution regions centered on the transcription start site (TSS), covering 5 kb upstream and downstream. Heatmaps distinguish genes bound specifically by H3K4me3, H3K27me3, and bivalent genes.

To further explore the impact of STAG2 on the naive-primed transition, we evaluated the expression of naive and primed gene sets in STAG2 KO cells compared to WT cells. Our analyses reveal that STAG2 deletion significantly activates naive gene expression while concomitantly downregulating primed gene expression (Fig. 3, *A* and B). Further examination of specific pluripotency marker genes through qPCR confirmed that the deletion of STAG2 leads to increased expression of the naïve marker gene *Nanog* and decreased expression of the primed marker gene *Lin28a* (Fig. 3*C*). Subsequent validation through Western blot analyses reinforces these observations, underscoring the positive regulatory role of STAG2 in the naive-primed transition process (Fig. 3*D*). Notably, the deletion of STAG2 promotes the maintenance of naive pluripotency in mESCs, as evidenced by the upregulation of naive marker genes and the downregulation of primed marker genes at both the transcriptional and protein levels. These findings collectively underscore the pivotal role of STAG2 in modulating the pluripotency state transition in mESCs.

### Depletion of STAG2 results in reduced bivalent gene count in mESCs

During embryonic development, mESCs must adeptly respond to differentiation cues while retaining their pluripotent state. This delicate balance is maintained, in part, by the presence of bivalent chromatin domains harboring both activating (H3K4me3) and repressive (H3K27me3) histone modifications at the promoter regions of lineage-specific genes. These genes, termed bivalent genes, play crucial roles in orchestrating lineage specification and differentiation processes. In naive mouse preimplantation embryos, the repertoire of bivalent genes is limited and dynamically regulated; however, their abundance substantially increases following implantation, corresponding to the transition to the primed state and reflecting their heightened importance in lineage specification mechanisms (31, 32).

To delve into the impact of STAG2 deficiency on bivalent gene dynamics, we conducted a comparative analysis of genes individually marked by H3K4me3 and H3K27me3, as well as those exhibiting both modifications, between wild-type (WT) and STAG2 knockout (KO) cells. Visualization of the gene distribution heatmap revealed a notable increase in the number of genes marked solely by H3K4me3 in STAG2-deficient cells, while the count of genes marked exclusively by H3K27me3 remained relatively unchanged. Notably, examination of bivalent genes, characterized by the presence of both H3K4me3 and H3K27me3 modifications at the same genomic loci, unveiled a discernible decrease in their abundance in STAG2 KO cells compared to the WT counterparts (Fig. 3, *E* and *F*). This observation underscores the role of STAG2 in modulating the bivalent gene landscape in the mESCs genome, aligning with previous results indicating its involvement in promoting the transition from a naive to a primed pluripotent state.

### STAG2 facilitates the expression of primed marker genes during differentiation in mESCs

**Figure 4:**
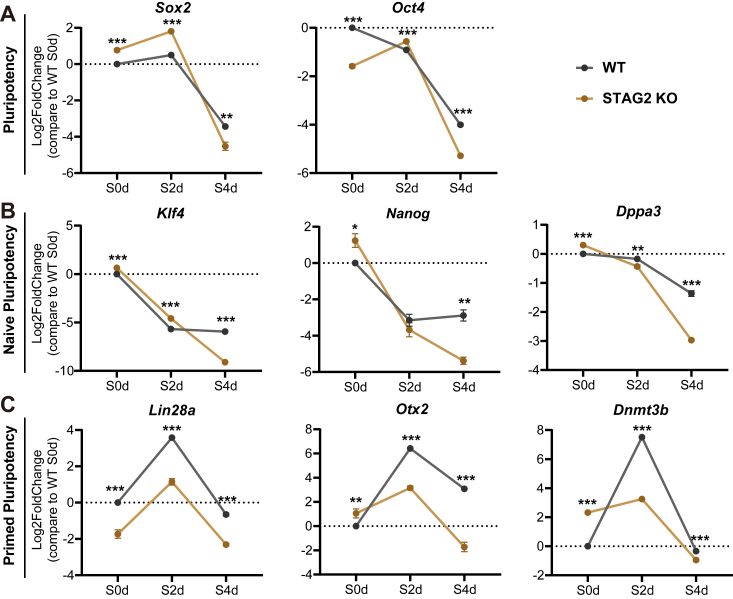
**STAG2 depletion reduces primed marker gene expression during the naive-primed transition.** qPCR analysis results reveal that core pluripotency genes *Sox2* and *Oct4* (*A*), as well as naive marker genes *Klf4*, *Nanog*, and *Dppa3* (*B*), are minimally affected during differentiation. STAG2 loss notably hinders the increase in expression of primed marker genes *Lin28a*, *Otx2*, and *Dnmt3b*, especially *Lin28a*, which remains significantly lower than WT after STAG2 deletion (*C*). The x-axis denotes three time points: S0d (naive pluripotent state), S2d (primed pluripotent state), and S4d (differentiation state). The y-axis represents relative mRNA levels compared to the WT S0d group, shown as log2-fold changes *via* qPCR (2∧(-ΔΔCt) values). Data represent the mean of three replicates, with error bars indicating standard deviations (not shown if too small). Statistical significance was assessed using two-tailed t-tests: ∗∗∗*p* < 0.001, ∗∗*p* < 0.01, ∗*p* < 0.05.

To further investigate the mechanism by which STAG2 deficiency impacts the naive-primed transition, we employed qPCR to assess the transcriptional levels of core pluripotency genes, as well as naive or primed marker genes, across key time points of differentiation: S0d (naive pluripotency state), S2d (primed pluripotency state), and S4d (differentiation state). Our experimental findings revealed that STAG2 depletion minimally influenced the overall expression trends of core pluripotency genes *Sox2* and *Oct4* during differentiation (Fig. 4*A*). Regarding naive marker genes *Klf4*, *Nanog*, and *Dppa3*, while STAG2 deficiency resulted in their upregulation in the naive state (S0d), this upregulation was insufficient to counteract the differentiation trajectory, as evidenced by a significant decrease in naive marker gene expression during differentiation, mirroring the trend observed in wild-type cells (Fig. 4*B*).

Conventionally, primed marker genes exhibit an initial upregulation followed by downregulation during differentiation. However, our results indicate that the loss of STAG2 imposes a substantial limitation on the upregulation of primed marker genes during the naive-primed transition at S2d. Specifically, the expression levels of *Lin28a*, *Otx2*, and *Dnmt3b* in STAG2 knockout cells at S2d significantly deviated from those in the wild-type group (Fig. 4*C*). Collectively, these findings suggest a positive regulatory role for STAG2 in driving the naive-primed transition by facilitating the upregulation of primed marker genes during the differentiation process.

### STAG2 facilitates the naive-primed transition of mESCs by enhancing Lin28a transcription and cytoplasmic localization

**Figure 5:**
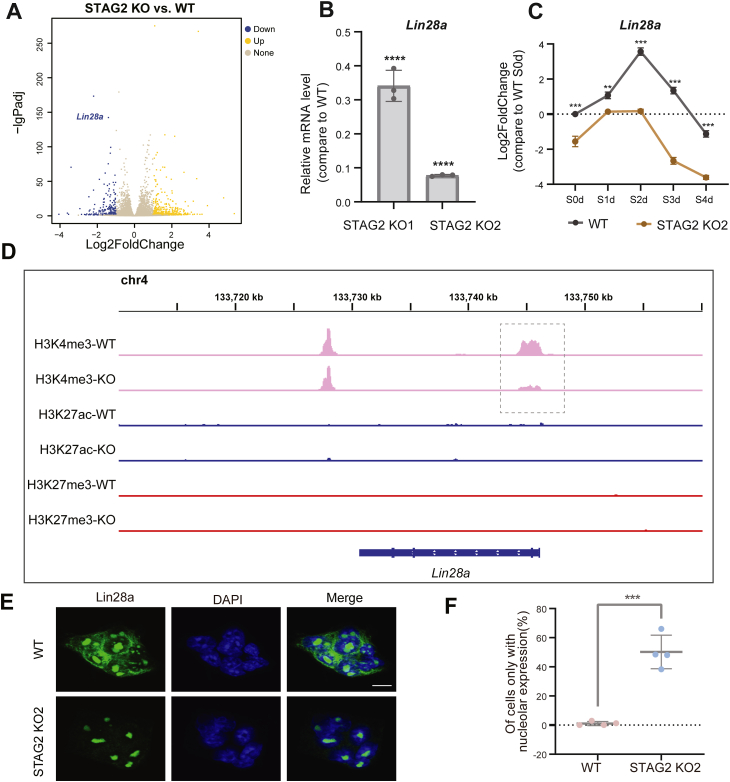
**STAG2 depletion significantly reduces Lin28a expression and alters its cytoplasmic localization.***A*, volcano plot of differentially expressed genes in STAG2 KO cells compared to WT, highlighting *Lin28a* downregulation. *B*, qPCR analysis confirms reduced *Lin28a* mRNA levels in STAG2 KO cells. *C*, qPCR results reveal that during differentiation, *Lin28a* expression remains significantly lower in STAG2 KO cells. *D*, enrichment peaks of histone modifications near the *Lin28a* gene suggest altered transcriptional activation due to STAG2 loss. The dashed box refers to the H3K4me3 enrichment near the transcription start site. *E*, immunofluorescence reveals predominant nucleolar localization of Lin28a in STAG2 KO cells. *F*, quantification confirms increased nucleolar localization of Lin28a in STAG2 KO cells. Four experiments were conducted for WT and STAG2 KO cells, with two cell lines for STAG2 KO1/2. Total cell counts: WT group (n = 142, 67, 62, 75), STAG2 KO group (n = 126, 66, 47, 52). Error bars indicate standard deviations. Statistical significance was determined using two-tailed t-tests: ∗∗∗*p* < 0.001.

To elucidate the molecular mechanisms underlying STAG2-mediated promotion of the naive-primed transition, we conducted RNA sequencing analysis to compare gene expression profiles between STAG2 knockout (KO) and wild-type (WT) cells. Our analysis revealed a notable downregulation of *Lin28a* transcription in STAG2-KO cells, with Lin28a being among the top five genes exhibiting the most significant differences and the sole pluripotency-related gene within this subset (Fig. 5*A*). To further validate the impact of STAG2 deficiency on *Lin28a* expression, we assessed *Lin28a* mRNA levels in two distinct STAG2 KO cell lines using qPCR. Our results demonstrated a substantial downregulation of *Lin28a* mRNA in both STAG2 KO cell lines compared to WT cells (Fig. 5*B*). Consistent with these findings, Western blot analysis corroborated the downregulation of Lin28a protein levels in STAG2-deficient cells (Fig. 3*D*). Moreover, we examined the transcriptional dynamics of Lin28a during differentiation from day 0 to four and observed a consistent downregulation of Lin28a expression in STAG2-KO cells throughout the entire differentiation process (Fig. 5*C*). Collectively, these findings underscore the significant impact of STAG2 deficiency on the downregulation of Lin28a expression.

To further explore the mechanism underlying STAG2 deficiency-induced downregulation of Lin28a, we employed CUT&Tag assays to assess histone modifications associated with transcriptional activity at the *Lin28a* gene locus in both wild-type (WT) and STAG2 knockout (KO) cells. Our findings unveiled a significant reduction in H3K4me3 marks within the *Lin28a* gene promoter region in STAG2 KO cells (Fig. 5*D*). This observation suggests that the absence of STAG2 may impede the transcriptional activation of Lin28a, consequently leading to a decrease in both its transcription and expression levels, thereby facilitating the naive-primed transition.

It has been reported that Lin28a, serving as a primed pluripotency factor, primarily modulates the levels of Dnmt3a/3b *via* let-7 in the cytoplasm, thereby regulating the transcription of naive marker genes (19, 33). Hence, we sought to investigate whether the loss of STAG2 influenced the cellular localization of Lin28a. Immunofluorescence experiments revealed that STAG2 deficiency resulted in the loss of cytoplasmic localization of Lin28a, with localization predominantly confined to the nucleolus (Fig. 5, *E* and F).

To elucidate the mechanism by which STAG2 deficiency leads to Lin28a depletion from the cytoplasm and its accumulation in the nucleolus, we considered the impact of Lin28a expression levels on its subcellular distribution. Given that STAG2 knockout diminishes Lin28a expression, we hypothesized that Lin28a expression levels might influence its cytoplasmic localization. To test this hypothesis, we employed RNA interference to selectively suppress Lin28a expression in mESCs. Our experimental findings indicate that as the expression level of Lin28a decreases, it initially loses its cytoplasmic localization and becomes retained primarily within the nucleolar region (Fig. S8, *A–C*).

To further investigate the molecular basis of Lin28a′s nucleolar localization, we conducted protein binding mass spectrometry, comparing Lin28a-interacting proteins in wild-type and STAG2-knockout mESCs. We enriched Lin28a-bound proteins using endogenous protein immunoprecipitation, with IgG as a negative control. Mass spectrometry analysis revealed significant interactions between Lin28a and various nucleolar proteins. Notably, while STAG2 deletion did not substantially alter these interactions overall (Fig. S8*D*), it enhanced Lin28a′s binding affinity for specific nucleolar proteins, including Nsa2, Rrp9, Eif3a and Rcl1.

Based on these findings, we conclude that Lin28a′s subcellular localization in mESCs is finely regulated by its expression level. Under conditions of low Lin28a expression, such as during cellular differentiation or induced repression, Lin28a preferentially interacts with nucleolar proteins (*e*.*g*., Nsa2) and localizes to the nucleolar region. Conversely, high Lin28a expression results in a partial cytoplasmic distribution of Lin28a proteins.

Previous studies have elucidated the temporal dynamics of Lin28a localization during embryonic development, wherein Lin28a expression transitions from nucleolar localization at the 8-cell STAG2 to expression in both the nucleolus and cytoplasm at the blastocyst STAG2. Subsequently, upon implantation, Lin28a becomes predominantly cytoplasmic (34). This nuanced spatial localization pattern suggests a correlation between nucleolar localization of Lin28a and naive pluripotency, while cytoplasmic localization is associated with primed pluripotency. The observed loss of cytoplasmic localization and retention of nucleolar localization of Lin28a in STAG2-deficient cells further substantiates the positive regulatory role of STAG2 in driving the naive-primed transition.

### STAG2 modulates Lin28a localization to govern mESCs pluripotency transition

**Figure 6:**
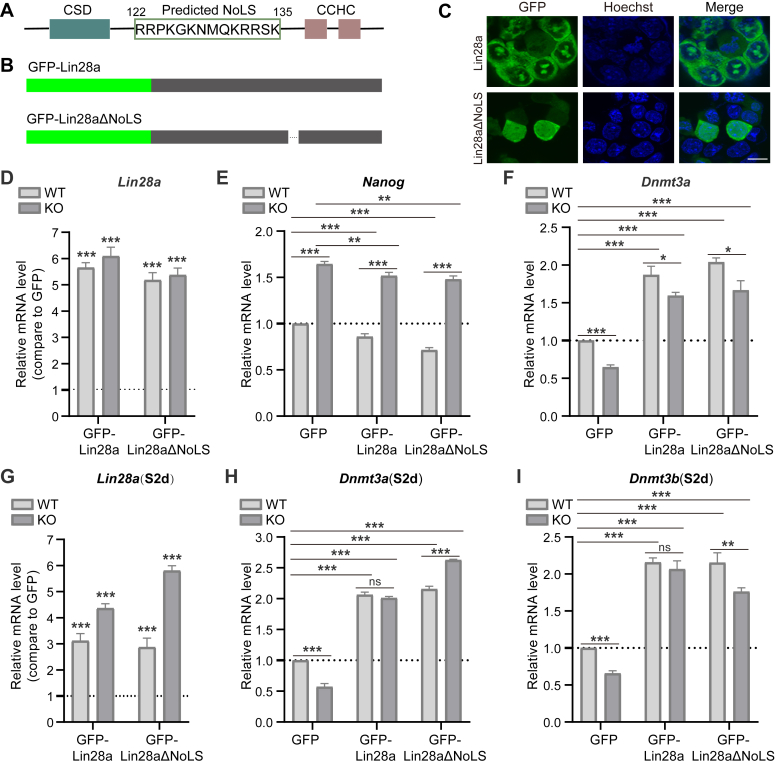
**STAG2 regulates the expression of primed marker genes Dnmt3a/3b *via* cytoplasmic Lin28a.***A*, Lin28a protein sequence features: Predicted nucleolar localization sequence (NoLS) indicated by *green box*, with position marked above. Flanking boxes represent characteristic domains CSD and CCHC. *B*, schematic diagram of full-length GFP-Lin28a and its truncated form without NoLS. *C*, live-cell imaging of GFP-Lin28a and GFP-Lin28aΔNoLS, while the latter showing distinct cytoplasmic localization and weaker diffuse nuclear localization, 48 h post-transfection. Scale bar: 10 μm. Hoechst used as live-cell DNA dye. *D–I*, qPCR detection of Lin28a and Lin28aΔNoLS overexpression effects on transcription levels of marker genes *Nanog*, *Dnmt3a*, and *Dnmt3b* in WT and STAG2 KO cells. Y-axis in *D* and *G* shows fold change in expression compared to GFP control. Y-axis in *E*, *F*, *H*, and *I* show relative expression levels in each group. Results are averages of three replicates; error bars indicate standard deviation. Two-tailed *t* test used for significance; ns = no significant difference, *p* > 0.05; ∗*p* < 0.05, ∗∗*p* < 0.01, ∗∗∗*p* < 0.001.

To delve deeper into the functional distinctions between nucleolar and cytoplasmic localized Lin28a, and to elucidate the impact of STAG2 deficiency on Lin28a, we identified the amino acid sequence RRPKGKNMQKRRSK as the nucleolar localization signal (NoLS) of Lin28a. Subsequently, we constructed a truncated GFP-Lin28aΔNoLS variant (Fig. 6, *A* and *B*). Upon transfection, we observed a complete loss of nucleolar localization in Lin28aΔNoLS, with localization predominantly confined to the cytoplasm and a faint, diffuse nuclear distribution (Fig. 6*C*).

Furthermore, we transfected both wild-type (WT) and STAG2 knockout (KO) cells with GFP-Lin28a and GFP-Lin28aΔNoLS plasmids to assess their effects on pluripotency factor expression levels at S0d (naive) and S2d (primed) time points. Our results revealed that overexpression of Lin28a or Lin28aΔNoLS led to a significant downregulation of Nanog expression at S0d (Fig. 6, *D* and *G*). Notably, the upregulation of Nanog expression induced by STAG2 deficiency was only partially rescued by overexpression of Lin28a or Lin28aΔNoLS (Fig. 6*E*), suggesting that Lin28a, particularly cytoplasmic Lin28a, may represent one but not the sole downstream mediator of STAG2.

Moreover, overexpression of Lin28a or Lin28aΔNoLS in WT cells resulted in a significant increase in *Dnmt3a* expression at S0d. Conversely, STAG2 deficiency led to a marked decrease in *Dnmt3a* expression, a deficit that was effectively rescued by overexpression of Lin28a or Lin28aΔNoLS in STAG2 KO cells (Fig. 6*F*). This finding underscores the role of STAG2 in promoting Lin28a transcriptional activation, thereby enhancing the naive-primed transition through downstream regulation of primed marker genes.

At S2d, overexpression of Lin28a or Lin28aΔNoLS effectively rescued the downregulation of *Dnmt3a/3b* expression induced by STAG2 deficiency (Fig. 6, *H* and I). These results collectively underscore the pivotal role of cytoplasmic Lin28a in this process. Collectively, STAG2 deficiency leads to downregulation of Lin28a, particularly cytoplasmic Lin28a, which subsequently downregulates the expression of primed marker genes *Dnmt3a/3b*, thereby maintaining the naive pluripotency state of mESCs. This finding further underscores the crucial role of Lin28a in STAG2-mediated regulation of the naive-primed transition.

**Figure 7:**
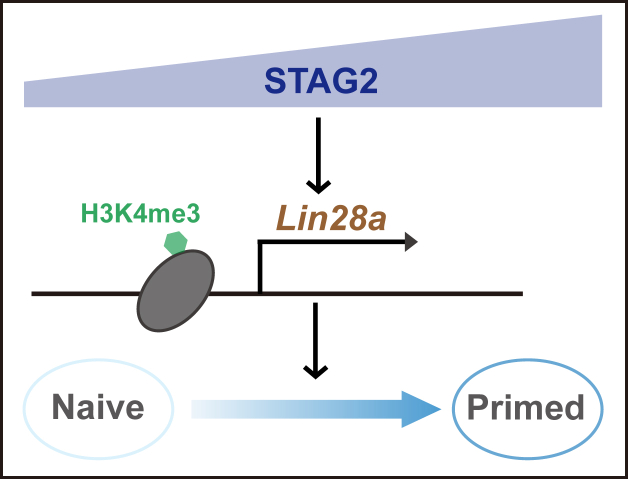
**STAG2 positively regulates the naive-primed transition in mESCs by promoting H3K4me3-mediated Lin28a transcriptional activation.** The expression level of STAG2 increases gradually during the naive-primed transition of mESCs, and it promotes the naive-primed transition of mESCs by facilitating H3K4me3-mediated transcriptional activation of Lin28a.

According to the findings delineated above, we proposed a functional model (Fig. 7) Within the context of the naive-primed transition in mESCs, there is a gradual escalation in the expression of STAG2. This upregulation subsequently fosters the transcriptional activation of Lin28a *via* heightened H3K4me3 levels. Consequently, these alterations in Lin28a expression and cellular localization culminate in the realization of the naive-primed transition within mESCs.

## Discussion

Exploration into the regulatory mechanisms governing the transition from naive to primed states in embryonic stem cells (ESCs) has been a focal point within the stem cell research field. Throughout differentiation, mESCs notably manifest a prolonged G1 phase. However, elucidating the precise molecular mechanisms that orchestrate pluripotency state transitions *via* cell cycle regulators has remained elusive.

In this study, we initially employed transcriptome sequencing to scrutinize the transition of mESCs from a naive to a primed pluripotency state, revealing significant alterations in the cell cycle dynamics during this developmental process. Subsequent investigations unveiled distinct expression patterns within two core subunits of the Cohesin complex, STAG1 and STAG2, throughout the naive-primed transition. Specifically, while STAG1 protein levels demonstrated a decline, those of STAG2 exhibited an increase. These findings suggest that STAG1/2 may wield contrasting regulatory influences over the naive-primed transition of mESCs.

Moreover, our investigations revealed that deficiency in STAG2 facilitated the activation of naive gene sets while concurrently suppressing primed gene sets. This perturbation was accompanied by overarching shifts in the transcriptional landscape of the mESC genome and a reduction in the number of bivalent genes. STAG2 is a core subunit of the Cohesin complex, which plays a pivotal role in chromatin architecture and gene expression regulation. The loss of STAG2 likely compromises the normal function of the Cohesin complex, thereby altering chromatin organization and, consequently, gene expression patterns. Bivalent genes, characterized by the simultaneous presence of both H3K4me3 (an activation mark) and H3K27me3 (a repression mark), are poised for rapid activation or repression in response to developmental cues. The disruption of Cohesin function due to STAG2 loss could potentially modify the epigenetic landscape, leading to alterations in the expression profiles of these bivalent genes. The precise molecular mechanisms underlying this relationship between STAG2 deficiency, Cohesin dysfunction, and changes in bivalent gene expression remain to be fully elucidated. Further research is required to illuminate the intricate interplay between Cohesin-mediated chromatin organization and the regulation of bivalent gene expression in the context of STAG2 loss.

Subsequent analyses unveiled pronounced alterations in the expression profiles of primed marker genes, notably *Lin28a*, upon STAG2 deficiency. Complementary CUT&Tag assays provided corroborative evidence indicating that STAG2 deficiency attenuated the transcriptional activation of *Lin28a*. Notably, in cellular assays, STAG2 deficiency elicited a significant reduction in the cytoplasmic localization of Lin28a, with preferential retention of its nucleolus localization. It is imperative to note that the cytoplasmic localization of Lin28a holds pivotal significance in facilitating the naive-primed transition. Importantly, the overexpression of Lin28aΔNoLS or Lin28a effectively rescued the downregulation of primed marker genes, such as *Dnmt3a/3b*, induced by STAG2 deficiency.

At the genomic level, STAG2 is documented to localize at enhancers, promoters, and Polycomb domains, playing a pivotal role in modulating gene expression (32). Consequently, it is reasonable that STAG2 exerts its influence on the maintenance and transition of pluripotency states in mESCs by orchestrating the activation of key genes, such as *Lin28a*. On a cellular level, the subcellular localization of Lin28a emerges as a critical determinant of pluripotency status, with nucleolus localization being associated predominantly with naive pluripotency, whereas cytoplasmic localization correlates primarily with primed pluripotency. Remarkably, this study underscores the dynamic nature of Lin28a localization within the nucleolus, which undergoes periodic alterations throughout the cell cycle. Intriguingly, during mitosis, Lin28a protein forms aggregates *via* phase separation and exhibits chromosomal localization in a bookmarking manner (data not shown). These observations underscore a nuanced interplay between Lin28a and chromosomes, suggesting its regulation by the cell cycle, albeit the precise mechanisms remain elusive. Concurrently, the stimuli instigating the upregulation of STAG2 expression during the naive-primed transition, as well as the mechanisms through which STAG2 facilitates the cytoplasmic localization of Lin28a, represent areas warranting further elucidation.

In summary, the study delved into the impact of STAG2 on the regulation of pluripotency states in mouse embryonic stem cells (mESCs). It unveiled that STAG2 exerts a positive regulatory function specifically during the transition from naive to primed pluripotency states. Furthermore, the research identified Lin28a as a pivotal downstream factor modulated by STAG2. The deficiency of STAG2 was observed to profoundly disrupt the transcriptional activity of Lin28a, thereby perpetuating the mESCs in a naive pluripotent state. Additionally, the regulatory influence of STAG2 on pluripotency extends to modulating the transcriptional activity of numerous genes and reshaping the expression pattern of bivalent genes. These findings contribute valuable insights into the intricate role of STAG2 in regulating pluripotency in mESCs.

## Experimental procedures

### Cell culture

mESCs E14TG2a were cultured under two distinct conditions: serum-free and serum-containing. The serum-free medium comprised DMEM/F12, Neurobasal medium, N2 supplement, B27 supplement, Glutamax, NEAA, sodium pyruvate, penicillin/streptomycin, β-mercaptoethanol, LIF, and 2i (CHIR99021, and PD0325901). The serum-containing medium consisted of DMEM/F12 supplemented with 20% fetal bovine serum, Glutamax, NEAA, sodium pyruvate, penicillin/streptomycin, β-mercaptoethanol, and LIF. Additionally, a Serum/LIF medium with 2i was utilized for mESCs culture in this study. For clarity, these three culture conditions were denoted as NL2i (N2-B27/LIF/2i), SL (Serum/LIF), and SL2i (Serum/LIF/2i), and were subjected to further testing (see Fig. S1).

### Antibodies

The antibodies used for immunofluorescence were: Lin28a (CST8760), Sox2 (sc-365823), Klf4 (Abcam, ab214666), Oct4 (Abcam, ab18976), STAG2 (Abcam, ab201451), and STAG1 (Abcam, ab4457). All secondary antibodies were from Invitrogen. Both the primary and the secondary antibodies were diluted in DPBS containing 3% BSA at a ratio of 1:200. The antibodies used for Western blotting were: Klf4 (Santa Cruz, sc-393462), GAPDH (Proteintech, 60004-1-Ig), α-Tubulin (Abcam, ab7291), Oct6 (Abcam, ab259952), Nanog (Abcam, ab203919), and Otx2 (Abcam, ab183951). These antibodies were diluted in 3% non-fat milk at a ratio of 1:1000.

### Molecular cloning and RNA-seq

The pEGFP-Lin28a-C1 plasmid was constructed using Lin28a gene from E14TG2a cell cDNA. E14TG2a cells from different culture conditions and induction treatments (three biological replicates per group) were collected, flash-frozen, and sent to Beijing Novogene Co., Ltd for RNA library construction and sequencing. Similarly, STAG1/2 KO and wild-type E14 TG2a cells (three biological replicates each) underwent RNA extraction and were sent to Annoroad Gene Technology Co, Ltd for RNA library construction and sequencing.

### Flow cytometry

Cells were fixed in 75% ethanol and stored at −20°C overnight. For cell cycle analysis, cells were stained with propidium iodide (PI) after fixation and washing. For cell proliferation analysis, cells were treated with EdU before fixation and then stained with Click reaction liquid and PI. For fluorescent antibody staining, cells were incubated with a primary antibody overnight and then with a fluorescent secondary antibody for 1 to 2 h. Finally, cells were washed and analyzed using a flow cytometer.

### Real-time fluorescent Quantitative PCR (qPCR)

Primer design for the target gene was done using the NCBI website as per experimental requirements. The qPCR reaction system comprised 10 μl, including 1 μl of cDNA, 1 μl each of upstream and downstream primers, 2 μl of ddH2O, and 5 μl of 2 × Universal SYBR Green Fast qPCR Mix. Reactions were conducted in an eight-tube strip or 96-well plate with one 10 μl reaction per well. Each sample was run in triplicate. Reaction mixtures were prepared, added to wells, briefly centrifuged, and run on the Light Cycler 96 Instrument using the SYBR program for three-step amplification. Data were exported and processed using Roche Light Cycler 96 software.

### Gene knockout

Revive E14TG2a cells with the lowest passage number, ensure suitable density, and good condition for transfection. Follow the molecular cloning protocol to extract sgRNA plasmid targeting the gene for knockout. Transfect each sgRNA plasmid into two 100 mm culture dishes with 12 μg of plasmid per dish. Conduct flow cytometry 48 h post-transfection. Culture cells in a 96-well plate for approximately 10 days, then passage surviving clones to a new 48-well plate for further culture. Collect half of the cells for PCR analysis to determine mutation type. Verify knockout cells with at least two rounds of PCR amplification and sequencing. Utilize knockout cells with frame-shift mutations for subsequent experiments.

### Mass spectrometry analysis

For mass spectrometry sample preparation, collect and lyse the processed cell samples according to established immunoprecipitation protocols, then incubate the lysates with appropriate beads for protein capture. Perform gel electrophoresis, allowing samples to migrate approximately one-third of the gel's length. Throughout the procedure, wear a lab coat and mask to minimize keratin contamination. After electrophoresis, remove the gel, excise the upper loading well portion, and rinse with double-distilled water (ddH2O). Stain the gel with Coomassie Brilliant Blue, then rinse again with ddH2O and destain on a shaker for 3 to 4 cycles of 10 min each, until distinct protein bands are visible. Excise the protein bands of interest and perform trypsin digestion. Select an appropriate mass spectrometry instrument for protein identification. Analyze the obtained mass spectrometry data using Proteome Discoverer 2.2 software, comparing the results against the Mouse Reviewed Swiss-Prot database for protein identification and characterization.

### CUT&Tag library construction

Follow the manufacturer's instructions for the CUT&Tag Assay Kit (pAG-Tn5) for Illumina (RK20265). Use the Dual DNA Adapter 96 Kit for One-step DNA Lib Prep for library construction. Send the constructed DNA library to Annoroad Gene Technology Co, Ltd for sequencing analysis.

### Data analysis

Data analysis was performed using Photoshop, Illustrator, and GraphPad Prism software. At least three independent replicate experiments were performed, and two-tailed t-tests were used to determine significant differences. RNA-seq data was processed using trim_galore, HISAT2, and HTSeq to analyze gene expression levels. Differentially expressed genes were identified using DESeq2 with a fold change >2 and Bonferroni-corrected *p* value < 0.05. CUT&Tag data was processed using trim_galore, Bowtie2, and MACS2 to identify peaks, and visualized using IGV.

### siRNA knockdown of Lin28a

Two siRNA sequences were employed in this experiment: siRNA1, 5′ CACCTTTAAGAAGTCTGCCAA 3′, and siRNA2, 5′ CTCCCAGAAGCCCAGAATTGA 3′. Mouse embryonic stem cells (mESCs) were cultured in six-well plates until they reached a density of approximately 60%. Subsequently, siRNA transfection was performed as follows: 125 μl of DMEM/F12 medium was mixed with 15 μl of Lipo2000 and incubated for 5 min. Concurrently, 125 μl of DMEM/F12 medium was mixed with 10 μl of siRNA and incubated for 5 min. The two mixtures were then combined and incubated for 20 min before being added to the cell culture dish. The dish was gently agitated to ensure thorough mixing and then placed in the incubator. After 8 h, the cell culture medium was replaced once to minimize cellular toxicity. Cells were subjected to subsequent experimental analysis 48 h post-transfection.

## Data availability

All data are contained within the manuscript.

## Supporting information

This article contains supporting information.

## Conflict of interest

The authors declare that they have no conflicts of interest with the contents of this article.
