# Supplementary material for: STAG2 promotes naive-primed transition via activating Lin28a transcription in mouse embryonic stem cells
Source: J Biol Chem. 2024 Nov 5;300(12):107958. doi: 10.1016/j.jbc.2024.107958 (PMC11635655; doi:10.1016/j.jbc.2024.107958)
Supplement: Supporting Information [file mmc1.pdf]

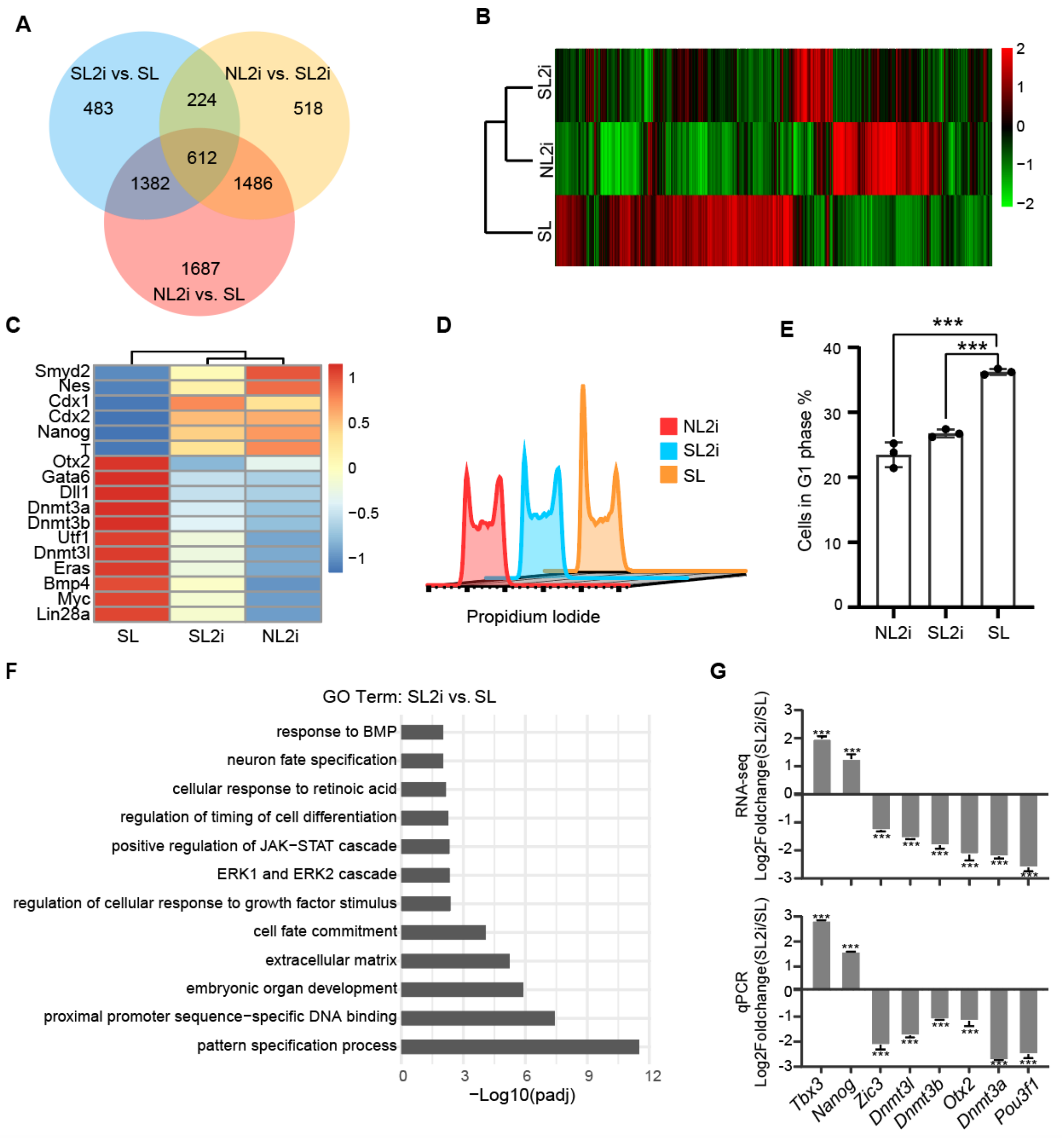

Figure S1 Adding 2i to the medium facilitate to establish a more-naive pluripotent state in mESCs. *A.* The Venn diagram illustrates the unique and overlapping differentially expressed genes across the different culture conditions. SL: Culture medium with Serum and LIF; SL2i: Culture medium with Serum, LIF, and 2i; NL2i: Culture medium with N2, B27, LIF, and 2i. *B.* Heatmap showcases differentially expressed genes across three culture conditions, clustered based on correlation. *C.* Heatmap highlights differentially expressed pluripotency genes under the three culture conditions. *D–E.* Flow cytometry analysis reveals cell cycle changes, notably an increase in G1 phase cells in the SL group. Statistical significance was determined using a two-tailed t-test (***P < 0.001). *F.* GO terms enriched by differentially expressed genes between SL2i and SL groups. *G.* Expression levels of naive and primed marker genes significantly differ between SL2i and SL groups. RNA-seq data and corresponding qPCR validation results are presented, with significant differences (Padj or P values < 0.001) indicated, along with standard deviation error bars.

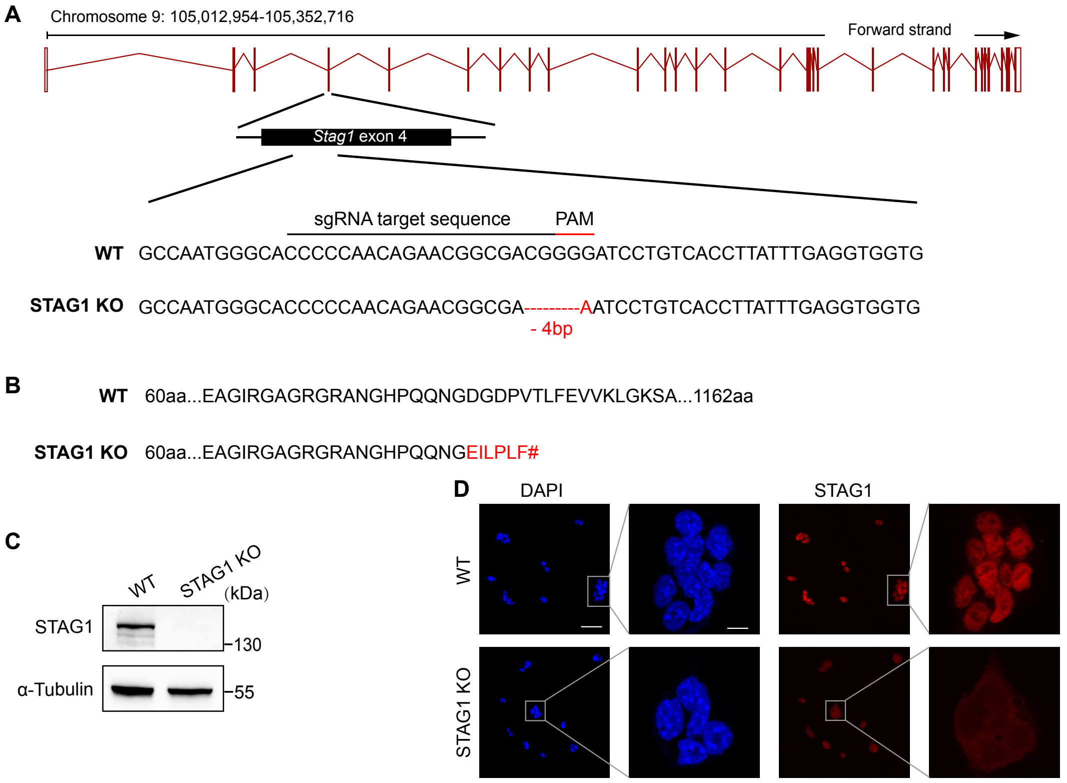

Figure S2 Construction of *STAG1* gene knockout cell line. *A*. Schematic diagram illustrating *STAG1* gene knockout on chromosome 9. The sgRNA targets the 4th exon of STAG1, with target and PAM sequences marked. Below is a partial gene sequence showing the deletion site. *B*. Protein sequence changes due to STAG1 knockout, with omitted amino acids marked. *C*. Western blot confirms complete knockout of STAG1 protein, with α-Tubulin as the loading control. *D*. Immunofluorescence shows disappearance of nuclear STAG1 protein localization in STAG1 KO cell line, with DAPI staining nucleus. Scale bar: 50 µm in broad view, 10µm in narrow view.

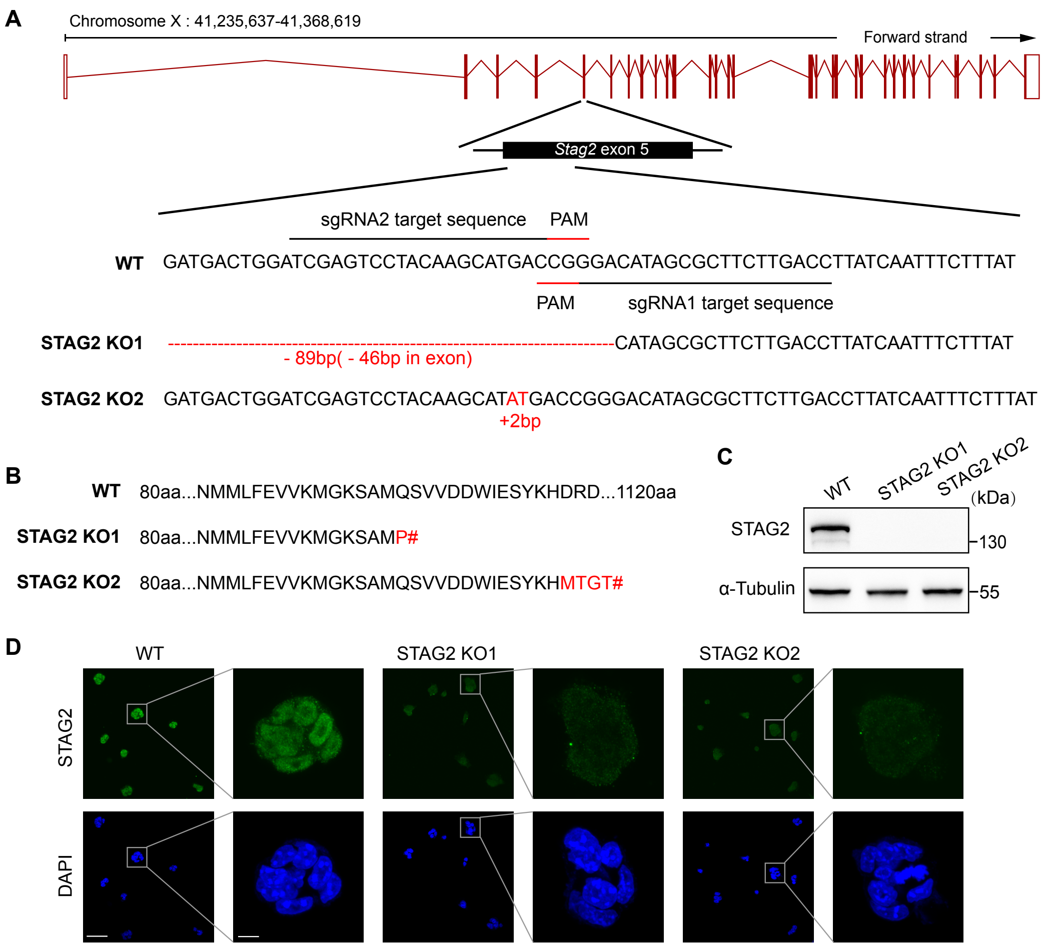

Figure S3 Construction of *STAG2* knockout cell lines *A.* Schematic diagram illustrating *STAG2* gene on the X chromosome. sgRNA1 targets the antisense strand, while sgRNA2 targets the sense strand, both focusing on the 5th exon. The sites of modifications are indicated in the STAG2 gene sequence, with deletions (KO1) and insertions (KO2) highlighted. *B.* Altered amino acid sequences due to STAG2 gene knockout are depicted, with red font indicating changes and '#' indicating termination. *C.* STAG2 protein knockout efficiency is demonstrated in two STAG2 KO cell lines by Western Blot analysis, showing complete elimination. α-Tubulin serves as the loading control. *D.* Nuclear localization of STAG2 protein disappears in STAG2 KO cell lines, as shown by immunofluorescence. Cell nucleus was stained with DAPI. Scale bar: 50 μm in broad view, 10μm in narrow view.

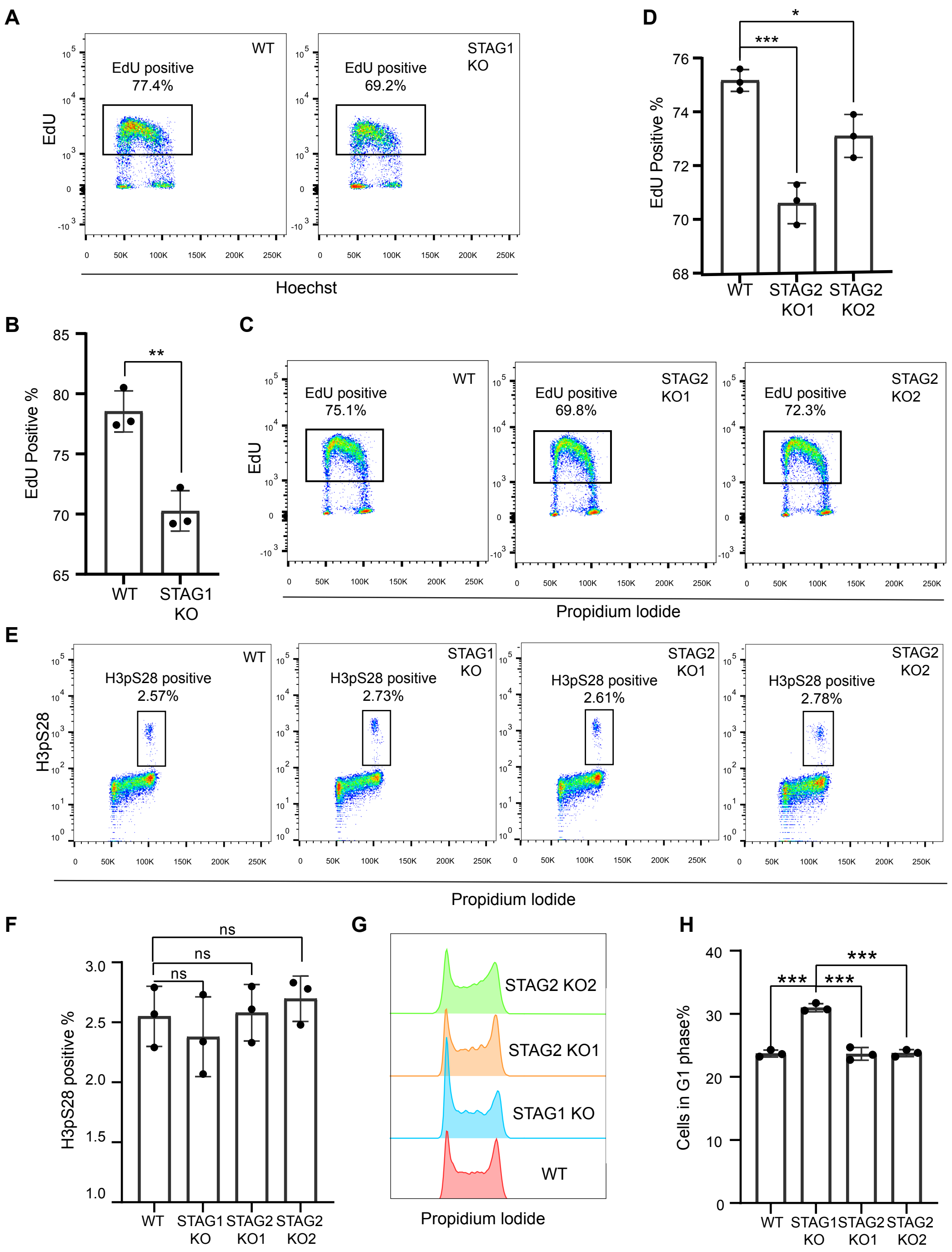

Figure S4 Effect of STAG1 or STAG2 loss on mESCs cell cycle. *A–D*. Both STAG1 and STAG2 Loss reduced the portion of S Phase. Decreased proliferation rate shown by EdU-positive cell proportion. *E–F*. Proportion of Cells in M Phase. No significant effect was observed. Statistical significance indicated by "ns" (P > 0.05). *G–H*. Flow Cytometry Analysis shows that loss of STAG1 increases G1 phase cells significantly. Statistical significance is indicated. All Statistical significance determined by two-tailed t-test (*P < 0.05, **P < 0.01, ***P < 0.001). Error bars represent standard deviation.

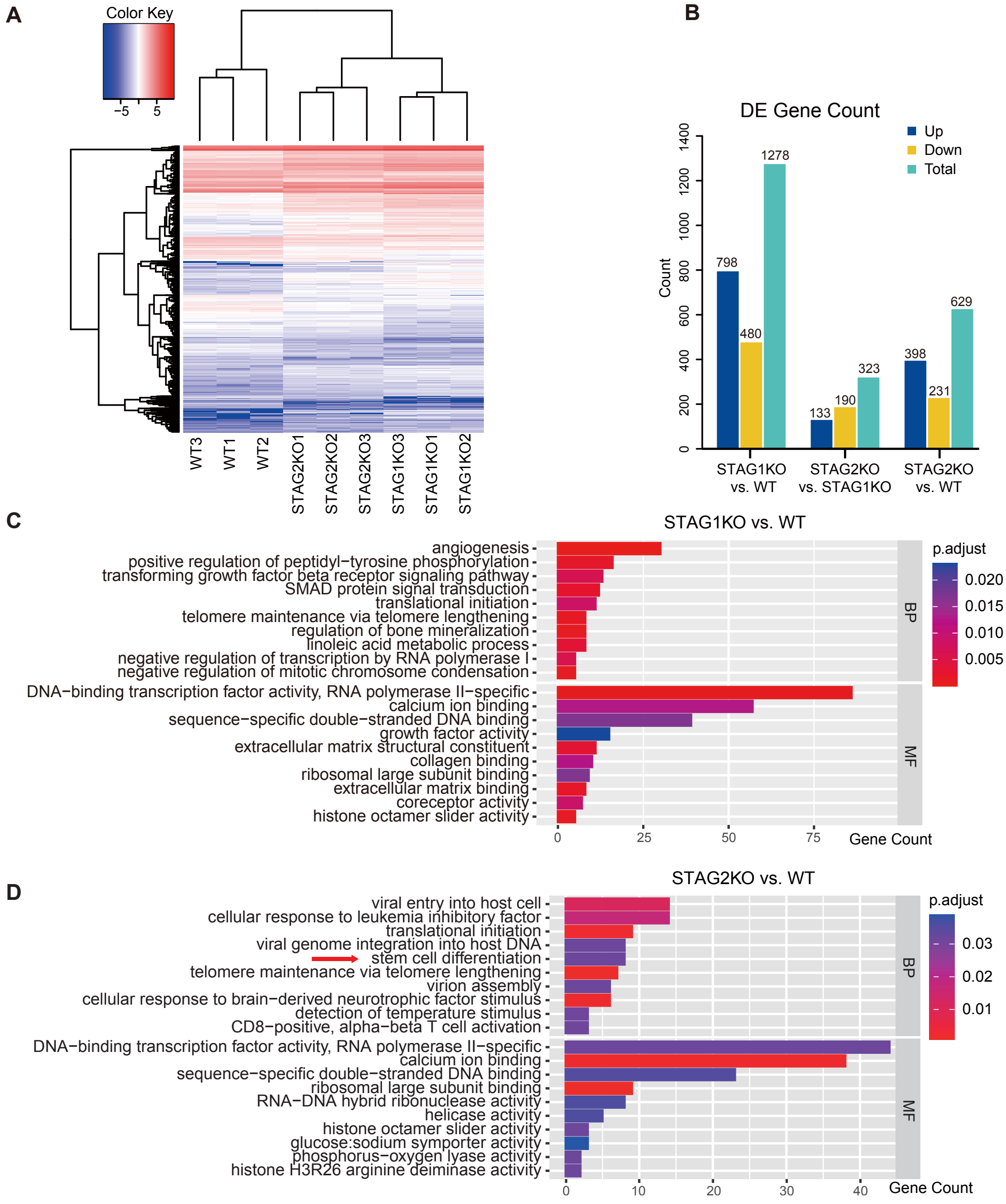

Figure S5 ***STAG1*** **or** ***STAG2*** **loss impact on gene expression and pluripotency.** *A.* Heatmap of Differentially Expressed Genes. Clustering among *STAG1* KO, *STAG2* KO, and WT samples, each with three replicates, indicating sample and gene clustering. *B.* Number of Differentially Expressed Genes. Pairwise comparisons among WT, *STAG1* KO, and *STAG2* KO samples, with blue for upregulated genes, yellow for downregulated genes, and teal for total differentially expressed genes. *C.* GO Term Enrichment. Top ten significantly enriched biological processes (BP) and molecular functions (MF) in *STAG1* KO samples compared to WT. *D.* GO Term Enrichment. Top ten significantly enriched BP and MF in *STAG2* KO samples compared to WT, with red arrows indicating pluripotency-related terms.

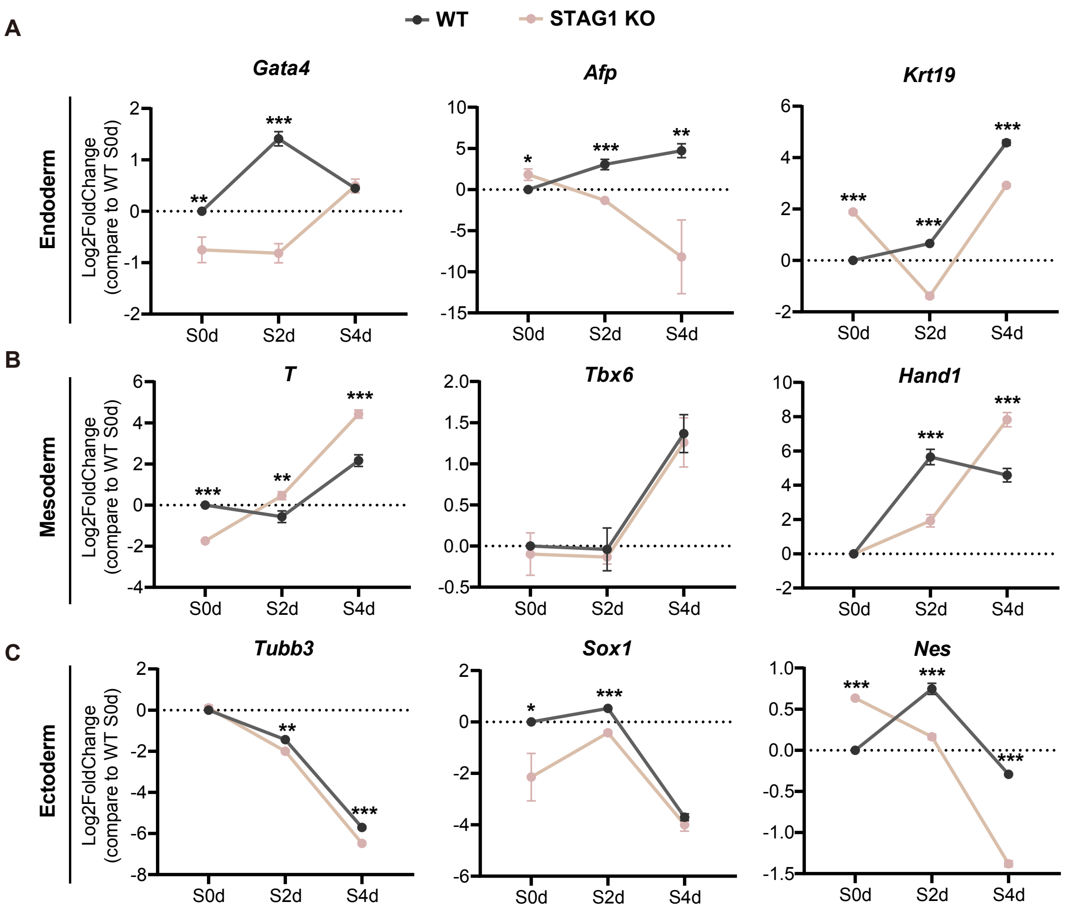

Figure S6 *STAG1* absence impacts endoderm differentiation in mESCs. The depletion of STAG1 protein modifies the expression levels of lineage-specific markers during mESCs differentiation, notably disrupting endoderm differentiation. *A.* The endoderm markers *Gata4*, *Afp*, and *Krt19* exhibit significantly decreased expression compared to WT at different time points throughout the differentiation process. *B.* The mesoderm markers *T* and *Hand1* are upregulated relative to WT, while *Tbx6* remains unaltered. *C.* Among the ectoderm markers, only *Nes* shows downregulation in later stages compared to WT, with *Tubb3* and *Sox1* experiencing minimal effects. The time points analyzed are S0d (naive pluripotency), S2d (primed pluripotency), and S4d (differentiated state). The vertical axis depicts the fold change in mRNA levels relative to the WT S0d group, presented on a logarithmic scale using $2^{-(\Delta\Delta Ct)}$ values. The left side of the figure denotes the three germ layers: endoderm, mesoderm, and ectoderm (from top to bottom). Each data point represents the mean of three replicates, with error bars indicating the standard deviation (omitted if smaller than the data point symbol). Statistical significance was assessed using a two-tailed t-test: ***$P < 0.001$, **$P < 0.01$, *$P < 0.05$.

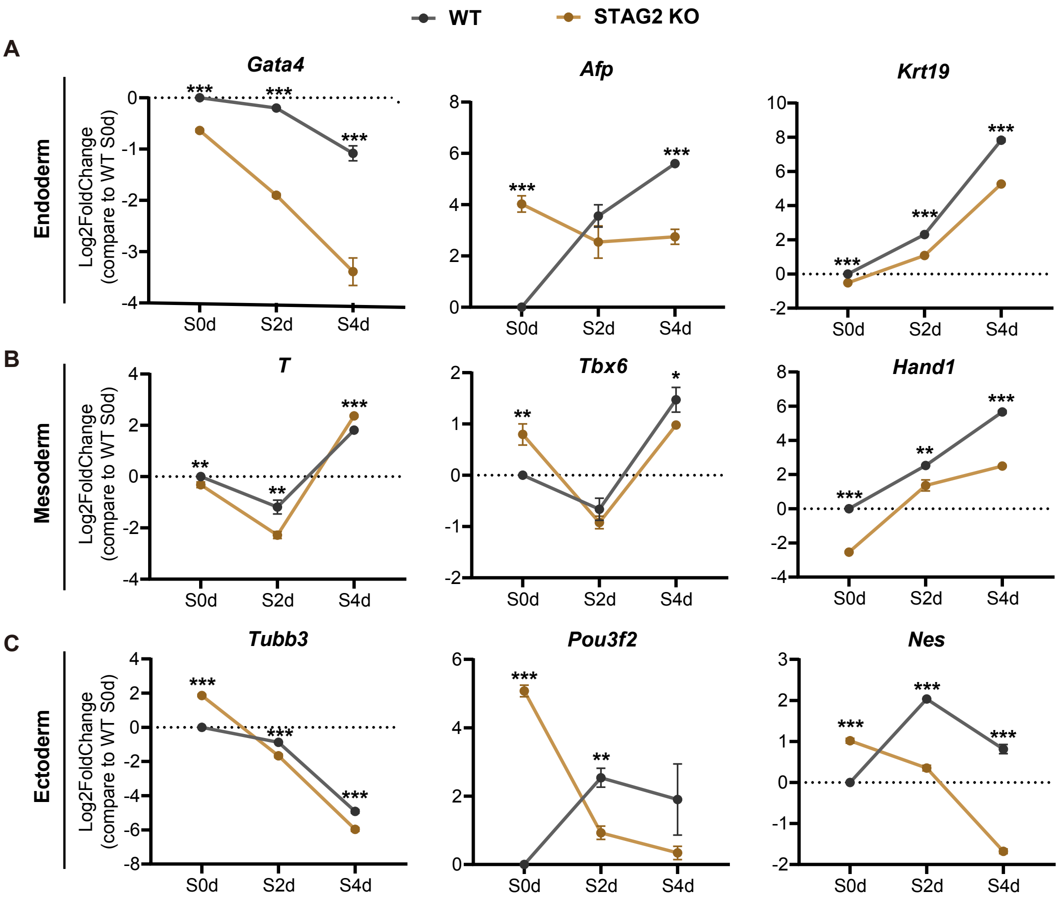

Figure S7 The Absence of *STAG2* impacts mESCs lineage differentiation. The depletion of *STAG2* influences the differentiation of mESCs into the three germ layers. A, Downregulation of endoderm marker genes (*Gata4*, *Afp, Krt19*); B, Downregulation of mesoderm marker genes (*T*, *Tbx6*, *Hand1*); C, Downregulation of ectoderm marker genes (*Tubb3*, *Pou3f2*, *Nes*) at different stages post-differentiation. These stages include S0d (naive pluripotency), S2d (primed pluripotency), and S4d (differentiated state). The vertical axis depicts the fold change in mRNA levels compared to the WT S0d group, presented on a logarithmic scale using $2^{-(\Delta\Delta Ct)}$ values. Each data point represents the mean of three replicates, with error bars indicating the standard deviation (omitted if smaller than the data point symbol). Statistical significance was assessed using a two-tailed t-test: ***P < 0.001, **P < 0.01, *P < 0.05.

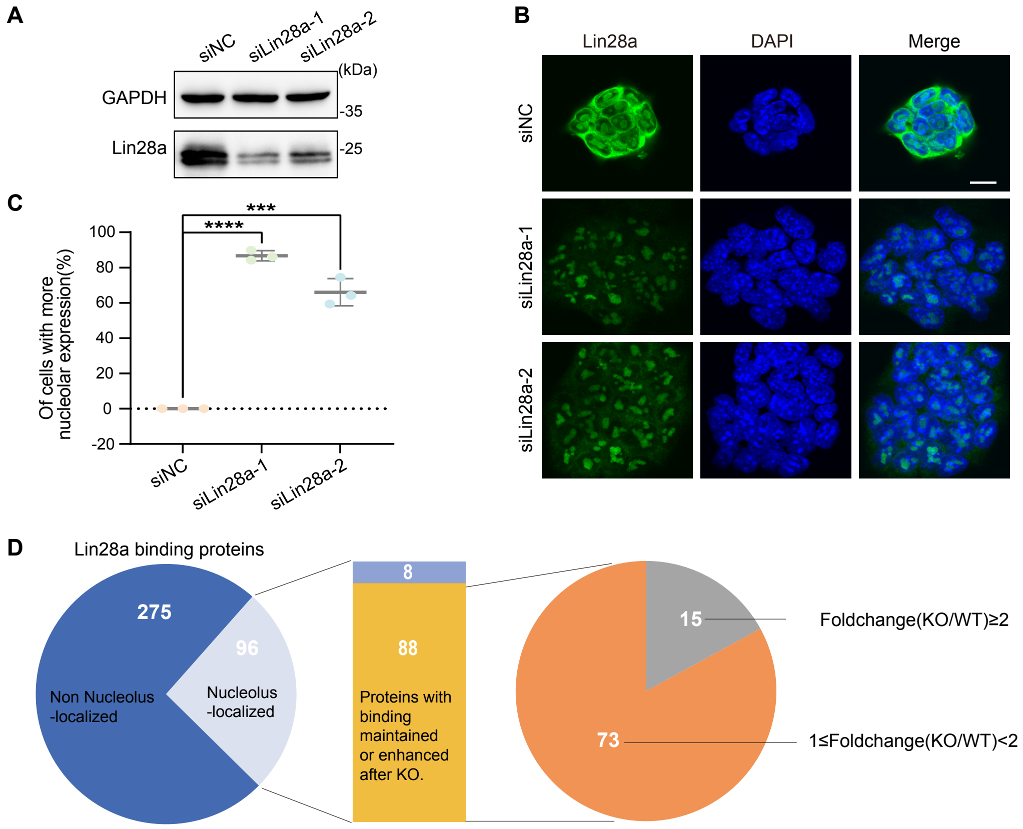

Figure S8 Lin28a Tends to Localize in Nucleoli After Knockdown. *A.* Western blot analysis of the interference effects of two siRNAs on Lin28a. *B.* Immunofluorescence detection of changes in Lin28a before and after knockdown in mESCs. After knockdown, the fluorescence intensity of Lin28a is significantly reduced, with prominent localization only in nucleoli. Scale bar: 10 µm. *C.* Statistics of the proportion of cells with Lin28a localized only in nucleoli in *B*. Three experiments were conducted for both the Negative control (siNC) group and the two siLin28a knockdown groups, with total cell counts of siNC (n=99, 96, 76), siLin28a-1 (n=128, 95, 129), and siLin28a-2 (n=137, 128, 102). Error bars represent standard deviations. Two-tailed t-test was used for statistical analysis, ***$P<0.001$. *D.* Mass Spectrometry Data Analysis: Comparison of peptide-spectrum matches (PSMs) between wild-type (WT) and negative control (NC) groups, using a fold change (F) threshold of $\geqslant 2$ to indicate effective interaction, yielded 371 data points. Cross-referencing these results with the GO:0007000 term in the Mouse Genome Informatics (MGI) identified 275 non-nucleolus-localized proteins and 96 nucleolar proteins. PSMs from STAG2 KO and WT groups were compared to categorize protein interactions. Interactions with a fold change (F) between 1 and 2 ($1 \leqslant F < 2$) were classified as unchanged, while those with a $F \geqslant 2$ were considered enhanced. Of the 96 nucleolar proteins identified, 88 exhibited either unchanged (73 proteins) or enhanced (15 proteins) interaction with Lin28a following STAG2 KO.
